# Supplementary figures and images for: Increased blood meal size and feeding frequency compromise Aedes aegypti midgut integrity and enhance dengue virus dissemination
Source: PLoS Negl Trop Dis. 2023 Nov 1;17(11):e0011703. doi: 10.1371/journal.pntd.0011703 (PMC10619875; doi:10.1371/journal.pntd.0011703)

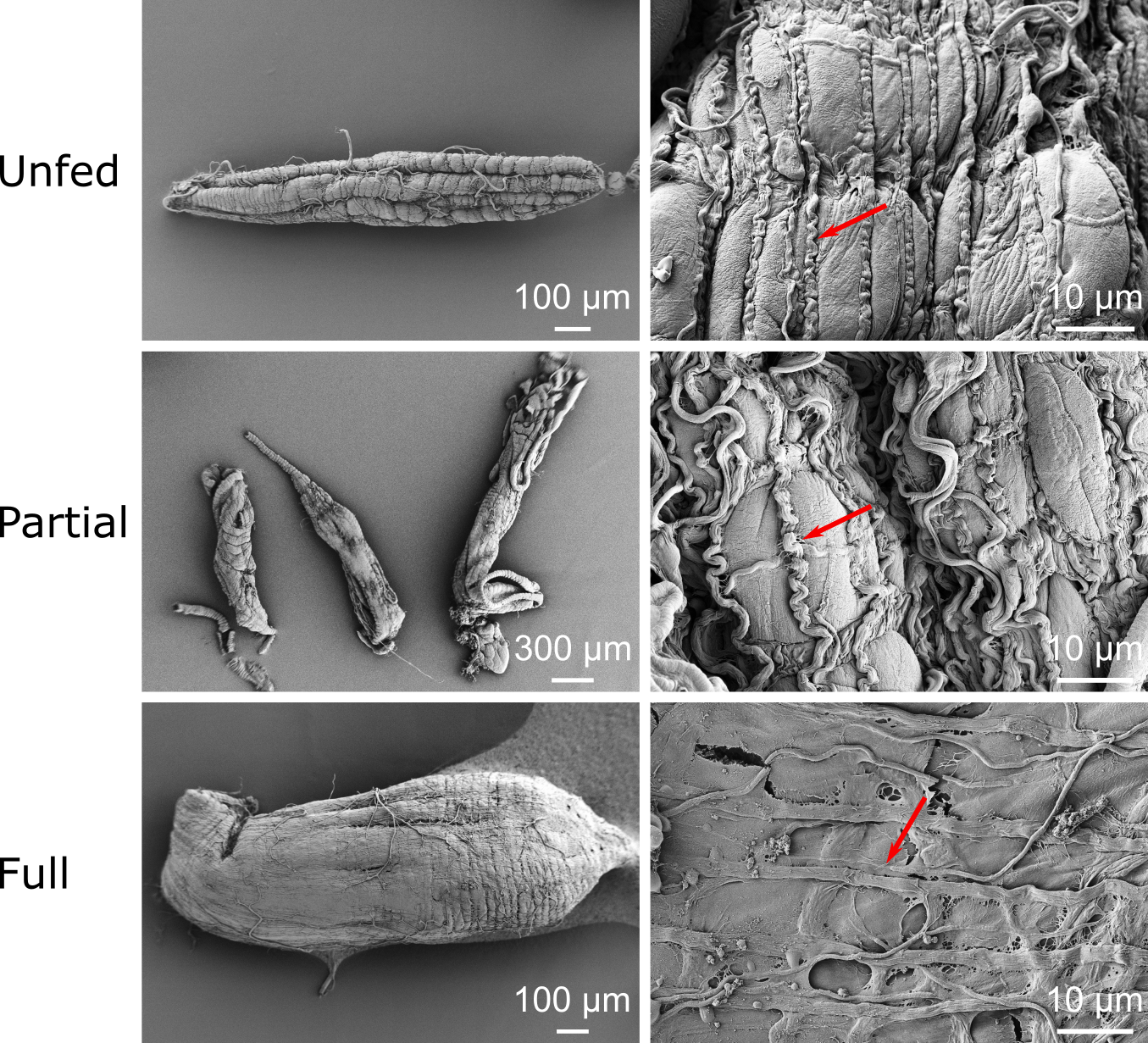

Supplement: S1 Fig — Overall midgut shape as well as stretching of muscles (arrows) surrounding midguts from mosquitoes given no blood meal (Unfed), a partial blood meal (Partial), and a full blood meal (Full). (TIF) [file pntd.0011703.s001.tif]

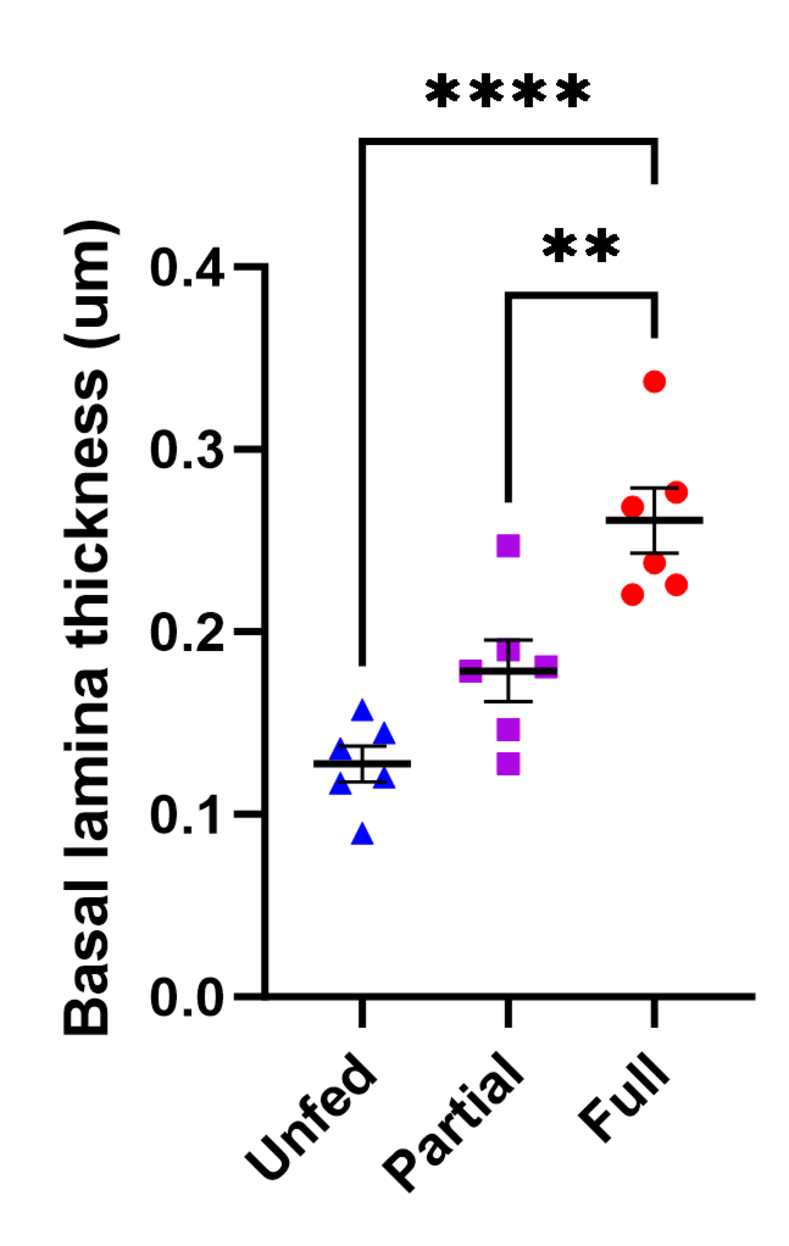

Supplement: S2 Fig — Basal lamina thickness 24 hpbm measured in areas that were not close to surrounding muscles. Measurements were from 2 mosquitoes per treatment with 3 TEM images per mosquito and 5 measurements per image that were averaged for each image to give 6 measurements per treatment (n = 6). Differences were assessed using a one-way ANOVA with a Tukey’s multiple comparisons post-test. On graph, center lines represent means and bars represent SEM. Exact p values for treatment comparisons are as follows: Unfed vs Partial p = 0.0776, Unfed vs Full p <0.0001, and Partial vs Full p = 0.0044. (TIF) [file pntd.0011703.s002.tif]
