# Supplementary material for: Increased blood meal size and feeding frequency compromise Aedes aegypti midgut integrity and enhance dengue virus dissemination
Source: PLoS Negl Trop Dis. 2023 Nov 1;17(11):e0011703. doi: 10.1371/journal.pntd.0011703 (PMC10619875; doi:10.1371/journal.pntd.0011703)
Supplement: S1 Text — (DOCX) [file pntd.0011703.s003.docx]

**S1 SUPPORTING INFORMATION**

**SEM imaging**

Midgut samples for SEM, were dissected 24 hpbm or at an equivalent time point if unfed. Dissections were performed in 0.1M cacodylate buffer with 4% paraformaldehyde (PFA) and samples were fixed in 2.5% glutaraldehyde, 2% PFA for 2 hours at room temperature before being placed at 4°C for 1-2 days. Midguts were rinsed with 0.1M cacodylate buffer and then processed by the Yale Center for Cellular and Molecular Imaging (CCMI) Electron Microscopy Facility. For each treatment group, midguts from three mosquitoes were submitted and imaged using a Zeiss Crossbeam 550 scope with a GEMINI II SEM column. Representative images were selected to show visceral muscle tension and overall midgut shape.

**Basal lamina thickness measurements**

Midgut TEM images of two mosquito midguts 24 hpbm or equivalent time point if unfed were acquired as described in the main text. From these, 6 TEM images per treatment (3 images per mosquito) were chosen and basal lamina thickness was measured in areas that were not close to surrounding musculature using the straight-line tool and measure function in ImageJ as described in the main text. Five measurements were taken per image and averaged for 6 measurements per treatment. Differences between treatments were assessed using a one-way ANOVA with a Tukey’s multiple comparisons post-test.
