# Supplementary material for: Increased blood meal size and feeding frequency compromise Aedes aegypti midgut integrity and enhance dengue virus dissemination
Source: PLoS Negl Trop Dis. 2023 Nov 1;17(11):e0011703. doi: 10.1371/journal.pntd.0011703 (PMC10619875; doi:10.1371/journal.pntd.0011703)
Supplement: Attachment — Submitted filename: Partial feed reviewer responses.docx [file pntd.0011703.s004.docx]

**Reviewer's Responses to Questions**

**Key Review Criteria Required for Acceptance? As you describe the new analyses required for acceptance, please consider the following:**

**Methods**

**-Are the objectives of the study clearly articulated with a clear testable hypothesis stated? -Is the study design appropriate to address the stated objectives? -Is the population clearly described and appropriate for the hypothesis being tested? -Is the sample size sufficient to ensure adequate power to address the hypothesis being tested? -Were correct statistical analysis used to support conclusions? -Are there concerns about ethical or regulatory requirements being met?**

**Reviewer #1: Additional details must be added to the methods regarding the high titer infections prior to publication.**

Response: Thank you for this comment. Upon further consultation with a former lab member, we discovered several errors in this section which have now been corrected. Specifically, minimally fed mosquitoes were also fed 1:5 diluted DENV-2, not concentrated DENV2 as previously reported. This has been corrected throughout the paper but changes are most extensive in the methods.

**Reviewer #2: The authors do not provide any reference for the method used to estimate the collagen hybridization assay they used. Also, they do not mention if the assay has been validated for insect tissues in general or for mosquitoes specifically. Also, it appears to me that this probe does not seem to be specific for collagen IV, but the authors assume that what they are measuring is only type IV. In the lack of fluorescence microscopy showing label is only at the BM, this statement may be just an hypothesis (although very likely).**

Response: The CHP peptide is not Col IV specific and binds to any form of damaged collagen. As we dissect out the midgut in these assays and expose only the exterior basement membrane (BM) side of the midgut to the CHP peptide, the majority of the collagen damage we are detecting is in the midgut BM and the major collagen type found in the BM is collagen IV. We have added in references that have used these same techniques in mosquitoes, included one where binding to the mosquito midgut is shown with fluorescent images (lines 163-167 reference 16). Additionally we have provided references to the development of these CHP probes (references 17 and 18).

**They do not give information on the amount of virus (PFU, preferably) used in the infection assays, what difficult comparison with data from the literature.**

**Also, in line 315 , they use a “high” concentration of DENV-2, but the reader receive no information on what they mean by high (or low) … The viral titers are evaluated only by qPCR, and not as infective particles, what is a weak point of the paper.**

Response: We made several changes in the methods section to clarify these protocols. As addressed above there was a mistake in the description of the minimal feeding experiments. These have been corrected throughout the paper but are most extensive in the methods section. The qPCR assays used in this paper were to assess positivity of bodies (infection) and legs (dissemination) and not viral titers as that was not the focus of this paper and previous data from Armstrong et. al., 2020 demonstrated no difference in infection intensity between mosquitoes fed a single blood meal vs. two blood meals (see Extended Data Fig. 4 from Armstrong et. al., 2020). Given these data, our focus was on the dissemination from the midgut and if this event took place or not. We have added infectious virus data for the blood and virus mixtures fed to mosquitoes in the form of focus forming assays. Titers from these ranged from 4.15x10^5-1.65x10^6 FFU/mL.

**Partial feeding (and minimal feeding as well) is observed qualitatively. This is ok for a routine protocol, but it would be useful for replication of their work by others if they quantify this partial as a range of blood volume (measured as weight, or hemoglobin or protein content compared to fully fed condition).**

Response: Thank you for this comment. We agree that our approach is qualitative, but for the purposes of this study we feel it is sufficient. We provide images in figure 1 which clearly show the different levels of engorgement used for each of the groups and all of our results are consistent with partial blood meals having an intermediate phenotype. Further, qualitative measures are routinely accepted in our field. The amount the mosquitoes imbibe is a relative measure and even fully fed mosquitoes differ in the amount of blood that they take in. For instance, no one questions whether or not a mosquito is fully engorged when reporting vector competence assays even though infection status could be affected by the amount of infectious blood imbibed. We were not attempting to perform a linear correlation between engorgement level and CHP damage, but rather we were trying to target a point between unfed and fully fed. For these reasons we do not feel that additional studies are warranted.

**Replication description is not clear in several parts of the paper. The major point here is to make clear the number of mosquitoes measured or the number of pools used. This is the true n.**

Response: Added in n values to captions of figures 1, 2, 3 and 4 where this was lacking.

**Fig 1 D, as an example, say thy collected 10 images per treatment, five measurements per image, but do not mention the number of insect replicates. In panel 1D, each point is a different pool of three mosquitoes?**

Response: We have added additional text to the methods section for clarity.

**Reviewer #3: (No Response)**

**--------------------**

**Results**

**-Does the analysis presented match the analysis plan? -Are the results clearly and completely presented? -Are the figures (Tables, Images) of sufficient quality for clarity?**

**Reviewer #1: Some small changes are needed in the figures to increase clarity.**

Response: Added in n values to all figure captions and moved error and center bars to the front so they are more visible.

**Reviewer #2: • They use qPCR just to say if the insect is infected or not. Of course, their raw data should include the amount of virus, what is a relevant information. This information could be made available, at least as a supplementary information.**

Response: We did not assess viral titer in insects but we have added in the titers fed to mosquitoes for experiments. For this study, titer was not a relevant measure for what we were interested in testing. We looked at infection and dissemination prevalence only. As mentioned earlier, it was previously found that second blood meals don’t impact viral titer. Armstrong et. al., 2020 demonstrated no difference in infection intensity between mosquitoes fed a single blood meal vs. two blood meals (see Extended Data Fig. 4 from Armstrong et. al., 2020). Given these data and our focus was on dissemination from the midgut, we did not quantify DENV2 titers in mosquito tissues. Clarified this part of the methods to make it clear that we were simply assessing presence of infection and dissemination.

**•	In Figure S2, while the bar size seems correct, the amplification apparently is about 10 X what should be. The bottom left photo certainly is not a 613 X magnification.**

Response: Thank you for this comment. Because there was an issue with uploading the supplemental figures, we believe this comment is actually about S1 (previously mislabeled as S2). We checked with the core that took these images and they say that current common practice is to exclude magnification but include size bars. As such we have removed incorrect magnification numbers from S2 and left in size bars.

**•	What is the red arrow in Figure S2? Structures present in the figure should be labelled.**

Response: There was a mistake in uploading the files and names were swapped. So S1 is the figure with arrows that point at musculature. See below caption.

“S1 Fig. Blood feeding leads to muscle stretching and may compromise gut integrity. Overall midgut shape as well as stretching of muscles (arrows) surrounding midguts from mosquitoes given no blood meal (Unfed), a partial blood meal (Partial), and a full blood meal (Full).”

**•	In several parts of the text the expression “infection levels “(ex: line 273) is used to refer to % of infection (prevalence). However, infection level is used more frequently to refer to amount of virus, so I suggest using this term for clarity.**

Response: Thank you for this comment. The reviewer is correct, and we have made the appropriate fixes throughout the manuscript when referring to infection and dissemination prevalence rates.

**•	Figure 2 legend says Midgut DENV-2 infection, but in methods only whole-body assays are mentioned**

Response: Examination of midgut and dissemination rates were determined by removing the legs of mosquitoes which would correspond to a disseminated infection. Because there cannot be a disseminated infection without there first being a midgut infection, we assayed the body minus the legs to establish midgut infection rates. If both body and legs are negative the mosquito is considered uninfected. If the legs are negative but the body is positive the mosquito is considered to have a midgut limited infection. Whereas both positive legs and bodies indicate a midgut infection and dissemination. The caption referenced doesn’t mention midgut infection. It only references midgut damage as assessed in 2B. We have added some language in Fig 2 and Fig 3 captions to make it clearer what tissues were examined. We have checked throughout the paper and altered fig 3 accordingly as this was an issue here and may be what the reviewer was referencing.

**Reviewer #3: (No Response)**

**--------------------**

**Conclusions**

**-Are the conclusions supported by the data presented? -Are the limitations of analysis clearly described? -Do the authors discuss how these data can be helpful to advance our understanding of the topic under study? -Is public health relevance addressed?**

**Reviewer #1: Yes**

**Reviewer #2: • I have two observations that I left to the authors to use or not, but I would like to share my thoughts on the subject with the authors. They look to CHP binding as an index of damage, and only in a single instance mention that this binding may be related to BM turnover. However, gut epithelia have probably the highest cellular turnover in most organisms. So, what they call damage, may also be described as use-related stress, followed by renewal. This would call the need not only for extracellular proteins (not only collagen) but also for cell death coupled to stem cell differentiation and division. Additionally, if we are talking about compromised barrier function of the gut upon distension, this may increase exposition of the body cavity to microbial elicitors, and trigger immune response that may lead to self-inflicted damage beyond the purely mechanical damage they assume in their model.**

Response: I don’t think this is mutually exclusive and we have discussed the turnover process the reviewer mentions in quite a few areas of the discussion. It seems likely that there is persistent turnover as discussed when talking about the CHP binding we still see in the absence of blood feeding but this turnover/damage/use-related stress increases with feeding, leading to increased CHP binding. In the discussion we talk about the possible mechanisms of repair/turnover more extensively and do mention the possibility of cellular involvement whether through stem cell proliferation or expression of MMPs. We agree with this comment but feel that the “speculative” part of the discussion mentioned below is critical for addressing these points raised here. We added in “turnover” and “stress” in a few places to make these points more clear but I think we are also just using slightly different words to describe the same processes. We have chosen to use “damage” whereas the reviewer suggests using “stress” and we have used “renewal” and “remodeling” whereas the reviewer prefers “turnover.”

**•	The discussion is too extensive and frequently recapitulates the results section. Some parts are too speculative, such as the one and half pages discussing SPARC function, for which there is no data in the paper.**

Response: See above. We don’t agree and think these speculative parts add to the overall model of gut turnover and repair in response to blood feeding. The 1.5 paragraphs that mention SPARC address several of the points Reviewer #2 brings up in the previous question and ties together what we’ve seen in our study with what is left to investigate further. Part of why this is slightly speculative is that the ECM has been more characterized in vertebrates but there is less known about ECM dynamics in insects outside of development or metamorphosis. We feel the discussion portion of the manuscript is a great place to discuss our work in the larger context and relate it to what is known throughout this field of research regardless if we have data directly addressing all of the specific topics brought up in this discussion.

**Reviewer #3: (No Response)**

**--------------------**

**Editorial and Data Presentation Modifications? Use this section for editorial suggestions as well as relatively minor modifications of existing data that would enhance clarity. If the only modifications needed are minor and/or editorial, you may wish to recommend “Minor Revision” or “Accept”.**

**Reviewer #1: Major Revision**

**Reviewer #2: In fig 1 B and D panels, the center lines should be placed in front of the data points in the D full data it is not possible to identify the position of the line because is it hidden behind the data points. There is no error bar visible in the figure, although it is mentioned in the legend.**

Response: Moved error bars and center bars in front of data points in all graphs.

**Reviewer #3: (No Response)**

**--------------------**

**Summary and General Comments Use this section to provide overall comments, discuss strengths/weaknesses of the study, novelty, significance, general execution and scholarship. You may also include additional comments for the author, including concerns about dual publication, research ethics, or publication ethics. If requesting major revision, please articulate the new experiments that are needed.**

**Reviewer #1: Comments to Authors**

**The manuscript by Johnson et al examine the effects of blood meal size, frequency, and repair of the midgut basal lamina in the context of arbovirus infection. The results are innovative and novel as they continue to explore the previously described effects of multiple blood meals on virus dissemination, providing new insight into the likely mechanisms of virus transmission in field settings where Aedes mosquitoes feed frequently after taking an infectious blood meal. The results are straight-forward and well-executed, such that the contributions will add a valuable piece to our understanding of mosquito vector competence. I have only a few comments which should improve clarity of the manuscript prior its publication.**

**Major Comments**

**-For the data in Figure 1D and Figure S1, it isn’t clear what data are displayed. If I understand correctly, there were 10 images with 5 measurements per image. It appears as though the authors display this as 50 points. I would argue this is artificial and unjustly increases the statistical power for these observations. I would argue that the repeat measures per image should be averaged, with data displayed for each of the 10 TEM images. This will likely change the statistical outcomes, but would argue this is a more accurate depiction of the limited data points.**

Response: We agree with this suggestion. We’ve updated this figure as well as S2 and methods and supplementary methods to reflect this approach.

**-The authors describe a “minimal blood meal” with concentrated virus, yet experimental details as to how this was performed are lacking in the methods. How do these “high titers” compare to regular infections? Infection titers should be included. This should be corrected for rigor and reproducibility.**

Response: Clarified this part of the methods as mentioned above and adding in titers quantified by focus forming assay. Titers from these ranged from 4.15x10^5-1.65x10^6 FFU/mL.

**-The authors present all of their infection data only as prevalence, without mention of viral titers. How does the infection intensity compare to prevalence?**

Response: We did not examine infection intensity as previous data from Armstrong et. al., 2020 demonstrated no difference in infection intensity between mosquitoes fed a single blood meal vs. two blood meals (see Extended Data Fig. 4 from Armstrong et. al., 2020). Given these data, our focus was on the dissemination from the midgut and if this event took place or not.

**Does a second blood meal (full or partial) influence infection? For transparency, all infection data should be included in the supplement as supplementary figures.**

Response: This data is presented in Figure 2C. Infection prevalence was not altered by a second blood meal either full or partial. The same percentage of mosquitoes became infected whether fed a single full blood meal or a full infectious blood meal followed by either a partial or full second blood meal. We did not assess infection intensity as previous data from Armstrong et. al., 2020 demonstrated no difference in infection intensity between mosquitoes fed a single blood meal vs. two blood meals (see Extended Data Fig. 4 from Armstrong et. al., 2020). The methods have been altered to clarify this (see below) and we’ve added a reference to this lack of connection between feeding and DENV titer.

**Minor Comments**

**-For Figure 1B, please display the number of pools examined in either the figure or figure legend.**

Response: Corrected this and followed previous comments above about averaging measurements by image.

**-In Figure 2C and 2D, it would be helpful to describe the tissues being evaluated. Based on 2A, it seems that this is the carcass (body) and legs being examined, but this is not explicitly stated in the figure or figure legend.**

Response: This is discussed in the methods but was added to the legend as well.

**-Figure 4A: The graphic shows harvesting body and legs similar that in Figure 2. However, I would argue that this is misleading since only midguts are being examined. Please correct.**

Response: We added in a midgut to the graphic and updated the within figure text to make this more clear.

**-For Figure 4B and 4C, I suggest respectively adding 24h and 96h to the figures to denote what the data represent without having to solely rely on the figure legend.**

Response: For consistency throughout the rest of the paper and figures we have left this as before. Timepoints for figures are described in the legends.

**-Figure S2: It is unclear what the arrows are pointing to. There is no mention in the figure legend.**

Response: This is due to a mistake in uploading the supplemental figures-also addressed below. What was uploaded as S1 should be S2 and vice versa. So S1 is the figure with arrows that point at musculature.

**Specific Comments**

**-Line 86-87: What is meant by “often obscured by common mosquito sorting practices”? Please elaborate and cite any relevant references.**

Response: This refers to sorting practices that often lump blood fed mosquitoes together regardless of degree of engorgement. Reworded for clarity.

**-Line 106-108: Please revise sentence “In singly fed mosquitoes” for clarity. Try to refrain from use of multiple adjectives in the same sentence (such as decreasing, beginning, lowering, etc.) to improve clarity.**

Response: We agree with this correction! Removed “singly fed” in several places listed below.

**-Line 401: There is mention of “in contrast to previous studies”, yet there are no citations as to which the authors might be directly referencing.**

Response: Citation #3 at the end of the sentence is the paper that we are referencing. This work is mentioned in more detail in the previous sentence, specifically that we see an increase in basal lamina thickness whereas they saw a decrease in basal lamina thickness. Changed wording slightly here for clarity.

**-Lines 434-438: I found these sentences confusing. Please revise for clarity.**

Response: Thank you for the comment. We have reworded these sentences for clarity.

**-Lines 478-483: Long run-on sentence. Revise for clarity.**

Response: Agreed. Reworded and broke up into more sentences.

**Reviewer #2: This paper is a follow-up for the previous report Armstrong et al. In the first communication they made an important contribution that was showing that a second, even non infective blood meal within a short interval enhances viral transmission, proposing that this was due to microperforations in the basal lamina. Here they add a relevant complement to that initial report, while showing that even partial blood meals were capable of that action. Overall, this is still a relevant contribution, pointing to novel variables that should be taken into account to evaluate vector competence under realistic field conditions.**

**Reviewer #3: In this manuscript, the authors have examined the effects of partial blood meals on Aedes aegypti midgut basal lamina integrity and on dengue virus dissemination. The authors have tested whether partial blood meals, where the mosquito has not fed to repletion, will enhance virus dissemination similar to what they previously published regarding full blood meals. They claim that this more accurately reflects the natural situation where mosquitoes often are not able to take full blood meals due to host defensive behavior. Using a collagen binding assay and TEM to assess damage to basal lamina, the findings indicate that larger blood meals have more damaging effects than partial blood meals, but that partial meals still cause an intermediate level of damage. These effects correlate to levels of DENV dissemination. Interestingly though, increased damage is not required for midgut escape, since some proportion of minimally fed mosquitoes that do not have more basal lamina damage than unfed mosquitoes still develop disseminated infection. Also, even in fully fed mosquitoes the damage appears to have been resolved by the time that DENV escapes the midgut. Taken together these results provide some additional insights into the mechanisms of arbovirus midgut escape. I have the following specific comments:**

1. **The title is overly broad. The authors have only examined one mosquito species and one arbovirus. Suggest changing to “Increased blood meal size and feeding frequency compromise Aedes aegypti midgut integrity and enhance dengue virus dissemination”**

Response: Changed the title to reflect this suggestion. “Increased blood meal size and feeding frequency compromise *Aedes aegypti* midgut integrity and enhance dengue virus dissemination”

2. **A weakness of the study is that the dose of virus fed to the mosquitoes is unknown. It is not clear why the virus stocks used to infect mosquitoes were not quantified by RT-qPCR. This makes it more difficult produce consistent results, both within the laboratory and in other laboratories who may want to repeat these experiments or use this approach.**

Response: To address this weakness we have performed focus forming assays on the infectious blood meals and presented the data in the revision. Titers ranged from 4.15x10^5-1.65x10^6 FFU/mL.

3. **Line 345, BM1 should be BM2**

Response: Thank you for catching this; it has been corrected.

4. **Line 393, the authors refer to “persistent extracellular matrix renewal as has been seen in humans”. It is my impression that all extracellular matrix is continuously being turned over, as it is constantly degraded and resynthesized. The authors should look into this in more depth. For example, see review article PMC3225943.**

Response: This was meant to simply be an example of ECM renewal, not to imply that persistent renewal is limited to humans. ECM renewal in insects is poorly characterized but is likely occurring persistently. We have reworded the sentence to read:

“This may be the result of nonspecific binding by CHP or may indicate that even naïve mosquitoes have an inherent baseline level of basal lamina collagen IV damage or that persistent extracellular matrix renewal results in detectable collagen IV damage (16).”

5. **The order of the two supplemental figures appears to be reversed compared to how they are described in the legends and text.**

Response: Thank you for catching this. The figures got mislabeled during uploading of the manuscript and problem has been fixed.

6. **Line 541, National Institutes of Health**

Response: Fixed!

**--------------------**
